# Photocatalytic upcycling of PET into methane, hydrogen and high-value liquid products

**DOI:** 10.1039/d5gc03562g

**Published:** 2025-11-12

**Authors:** Madeline Weisweiller, Adrian Ertl, Cornelia von Baeckmann, Anil Kumar Sihag, Christian M. Pichler, Freddy Kleitz, Dominik Eder, Alexey Cherevan

**Affiliations:** a Institute of Materials Chemistry, TU Wien Getreidemarkt 9/BC/02 1060 Vienna Austria alexey.cherevan@tuwien.ac.at; b CEST – Centre for Electrochemistry and Surface Technology Wr. Neustadt Viktor-Kaplan-Straße 2 2700 Wiener Neustadt Austria; c Institute of Applied Physics, TU Wien Wiedner Hauptstraße 8 1040 Vienna Austria; d Department of Functional Materials and Catalysis, Faculty of Chemistry, University of Vienna Währinger Straße 42 1090 Vienna Austria

## Abstract

The harmful effects of daily plastic use are increasingly evident, with most waste burned or landfilled, leading to the formation of microplastics that pollute the environment and the food chain. While the full impact remains unclear, photoreforming of plastics has emerged as a promising sustainable abatement method. This study demonstrates the commercial potential of P25 TiO_2_ towards photocatalytic upcycling of polyethylene terephthalate (PET) microplastics by systematic exploration of the effect of co-catalysts, reaction temperature and oxygen presence on the generation of solar fuels and high-value liquid products. We demonstrate that while neat P25 yields minimal H_2_ evolution, increasing the reaction temperature enhances its production significantly, and the addition of Pt further boosts H_2_ generation by four orders of magnitude, resulting in 15.35 µmol h^−1^ of H_2_ and apparent quantum yield (AQY) values up to 0.45%. On par with H_2_, we observe the generation of CH_4_ from the reaction mixture, which we conclude to originate directly from PET rather than hydrogenation reactions. Liquid-phase analysis reveals diverse photoreforming products, including acetic acid, oxalic acid, formic acid and ethanol, with selectivity influenced by catalyst composition and reaction conditions. The feasibility of large-scale application of the process is further validated through prolonged irradiation tests using solar-simulated light and an upscaled setup, which demonstrate remarkable AQYs reaching 0.84%. These findings suggest PET photoreforming as a promising route for producing solar fuels and valuable chemicals, paving the way for sustainable plastic processing and upcycling.

## Abstract 2

We systematically study the key process parameters of polyethylene terephthalate (PET) microplastic photoreforming. We uncover that not only hydrogen (H_2_) but also methane (CH_4_) is generated and provide valuable mechanistic insights into the process.

## Graphical abstract

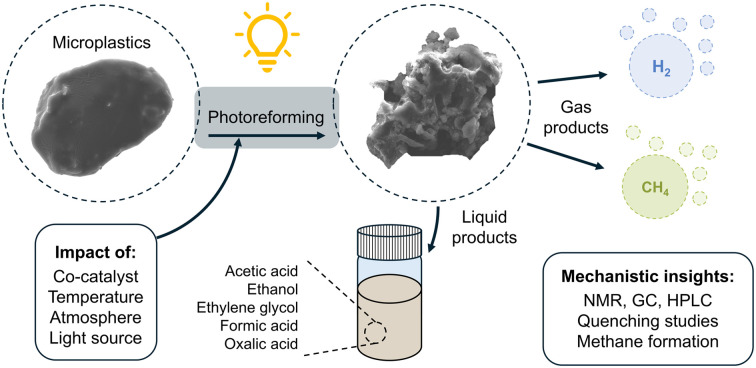

**Green foundation**

1. Our study demonstrates a significant advancement in photoreforming PET microplastics using P25 TiO_2_, transforming plastic waste into valuable solar fuels and chemicals, thus promoting sustainable waste management.

2. We achieved a notable boost in hydrogen generation by up to four orders of magnitude through the addition of Pt to P25 TiO_2_, resulting in H_2_ production rates of 15.35 μmol h^−1^ and AQY values reaching 0.45%. Additionally, we observed significant CH_4_ production and the conversion of PET into high-value liquid products such as acetic acid, oxalic acid, formic acid, and ethanol, demonstrating a viable route for converting plastic waste into valuable chemicals.

3. Future work should focus on optimizing the catalytic system to reduce noble metal use, exploring greener solvents, and conducting scalability studies to enhance sustainability and economic viability.

## Introduction

Plastics are ubiquitous materials, embedded in everyday life, bearing countless beneficial properties, such as light weight, cost-efficiency and aimed straightforward synthesis for various purposes.^1^ Although used for a plethora of applications and representing a great part of the world's economy,^2,3^ only a few abatement strategies are presently employed. A vast majority of plastic waste is simply discarded in landfills,^4^ gradually degrading to microplastic particles, down to 1000 nm in size.^5^ As a consequence, the environment and waters are contaminated^6^ and the full ramifications are yet to be elucidated.^7^ This issue has inspired the research community to investigate the applicability of photocatalysis for efficient conversion of microplastic, providing an opportunity to degrade the synthetic macromolecules and concurrently obtain green H_2_ – a sustainable fuel promising high energy densities without the worry of greenhouse gas emission^8,9^ – solely with the assistance of light. Initial success was already achieved in the early 1980s by Kawai and Sakata,^10–12^ converting saccharose, starch and cellulose to H_2_ with a mixture of RuO_2_/TiO_2_/Pt as a photocatalyst and in alkaline media.^11^ Their research was subsequently expanded exploring a wide range of organic substrates, including insect remains, polyvinyl chloride, excrements, algae and various carbohydrates. The employed TiO_2_/Pt catalyst showed promising results, yielding up to 1130 µmol of H_2_ in 10 hours.^10^ This now revisited concept, also known as photoreforming,^13^ has attracted a lot of interest in recent years, demonstrating successful photocatalytic valorisation of various organic compounds, such as cellulose^14–18^ polylactic acid (PLA)^19–21^ or polyethylene terephthalate (PET).^19,20,22–24^ A lot of work appeared involving TiO_2_ and its various polymorphs due to their commercial availability.^25–28^ Many insights into the photoreforming process have been provided by the group of Reisner *et al.* who investigated different visible light active photocatalysts, setups and substrates.^14,17,19,20,22,24,29^ In addition, a pretreatment methodology was established for an array of substrates ranging from microplastics to biomass constituents, oftentimes involving alkaline media, to enhance the degradation output and product yield of their studied systems.^19^ However, the use of high pH represents a bottleneck for currently applied materials, as it may interfere with the photocatalyst's long-term stability and would demand more resources.^13,30^ The extent of the impact of the strong basic conditions on the viability of the overall process is yet to be explored.

It is common that a synthetic macromolecule featuring polar bonds, such as PET or PLA, is selected as a model waste plastic, as these structures contain heteroatoms in the main chain that facilitate hydrolysis and subsequent upcycling.^31^ Although copious studies have investigated PET as a substrate,^28,32–36^ and thus provided a good base of knowledge on product formation and potential mechanisms, the current insights are still limited, as no systematic studies have been presented thus far. Furthermore, the focus of previous research works was often set on H_2_ production, rather than evaluating other gaseous products, such as CH_4_.^15,16,34^ A detailed investigation of PET photoreforming would further enhance our comprehension of this type of photocatalytic conversion, enabling new possibilities for its commercialisation. Product selectivity and the choice of the optimal, cost-efficient and sustainable visible light active photocatalyst, yielding high conversion outputs, are challenges that yet remain.^13^

While the majority of studies in this field of plastic photoreforming rather develop novel visible light active materials,^14,21,37^ we put our focus on the investigation of the very well-established TiO_2_ material.^38–41^ This photocatalyst has been studied for decades and provides a suitable model system, allowing us to investigate the process parameters and reaction mechanism. In the last few decades, numerous co-catalysts have been developed to address the challenges of direct water splitting.^42–45^ The use of such an auxiliary is also expected to promote photoreforming and result in higher H_2_ production rates.^46^ In a similar manner, increased temperature has shown to facilitate hydrolysis of the plastic source and affect catalytic rates,^47^ while control of the reaction atmosphere (aerobic *vs.* anaerobic) can be expected to impact reactive oxygen species generation and type. This study aims an in-depth exploration of each of these parameters on the photoreforming outcomes of PET microplastic powders exposed to 1 M NaOH and under UV irradiation. Our results highlight the beneficial effects of the noble metal co-catalyst Pt and elevated temperature on the formation of H_2_ and CH_4_. We further elucidate the potential origin of CH_4_ and examine the use of different light sources and process scales to provide a deeper understanding of underlying mechanisms and commercialisation prospects. Liquid phase analysis confirms successful photoconversion of ethylene glycol (EG) from PET to acetic acid (AA), oxalic acid (OA), formic acid (FA) and ethanol. This comprehensive investigation offers valuable insights to encourage implementation of this sustainable abatement strategy in the near future.

## Results and discussion

### Synthesis and characterisation

**Fig. 1:**
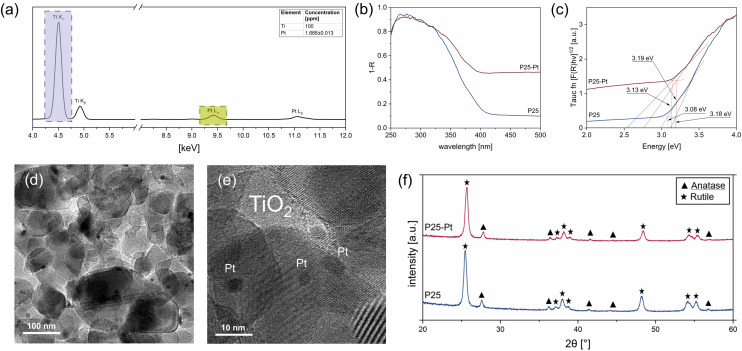
Characterisation of the prepared photocatalysts: (a) TXRF spectrum of P25-Pt confirming successful deposition of 1 wt% Pt onto P25, depicting the characteristic peaks for Pt (L_α_: 9.44; L_β_: 11.07) and Ti (K_α_: 4.51; K_β_: 4.93), (b) UV-vis DRS and (c) KM analysis of neat P25 and P25 with 1 wt% Pt as the co-catalyst, (d and e) TEM images of P25-Pt, and (f) XRD patterns of the P25 powder before and after Pt photodeposition.

The P25-Pt photocatalyst was obtained by photodeposition of commercial P25 powder with H_2_PtCl_6_ solution, aiming at 1 wt% of the noble metal loading (details in Experimental). After successful photodeposition, noticeable from a colour change from white to grey, the elemental composition of the photocatalyst was characterised *via* total X-ray fluorescence (TXRF) (Fig. 1a), revealing a Pt loading of 0.998 wt% with respect to TiO_2_. Scanning electron microscopy (SEM) (Fig. S3) and transmission electron microscopy (TEM) (Fig. 1d and e) analyses further demonstrate the prime particle size of TiO_2_ nanoparticles to be ∼24.9 nm and confirm the homogeneous distribution of Pt nanoclusters with an average size of 4.05 nm – typically expected from the photodeposition process from [PtCl_4_]^2−^ – on the titania surface.^48^ Diffuse reflectance spectroscopy (DRS) measurements and subsequent Kubelka–Munk (KM) analysis *via* the Tauc function^49^ (Fig. 1b and c) illustrate the expected absorption profile of P25, from which two band gap values of 3.08 and 3.18 eV – characteristic of the rutile–anatase mixed phase TiO_2_ – can be extracted. Loading of P25 with Pt nanoparticles results in continuous increase of its absorption profile throughout the UV-Vis region; however, virtually unchanged band gap values (3.13 and 3.19 eV, respectively) can be derived using a modified Tauc method, suggesting that the deposition did not affect the structure or optoelectronic properties of the supporting TiO_2_.^50^ The crystallinity of neat P25 and P25-Pt samples was investigated *via* powder X-ray diffraction (XRD) analysis (Fig. 1f). The diffractograms show virtually identical characteristic peaks of both anatase and rutile phases (89.3 : 10.7% for P25 and 90 : 10% for P25-Pt), whereas no additional peak related to Pt is visible in the pattern of P25-Pt expected from its low mass loading. We further note that Pt deposition on TiO_2_ leads to expected photoluminescence (PL) quenching (Fig. S2), which is commonly attributed to the created heterojunction at which the metallic Pt extracts photoexcited charges from the semiconducting support.

### Photoreforming experiments

The photocatalytic studies were carried out using P25 or P25-Pt as the photocatalyst and PET as the microplastic source. Both the catalyst powder and the model pollutant were first dispersed in 1 M NaOH to initiate cleavage of the polymer chain into EG and terephthalate (TP) blocks. The resulting suspension was next irradiated with a narrow band emitting LED source centred at 365 nm (details in Experimental and SI Note 2). In the course of the experiments, the temperature was either maintained at room temperature (RT, 25 °C) or increased to 70 °C. Furthermore, different reaction conditions were explored, assuming that an oxygen-rich (aerobic) atmosphere would expedite microplastic oxidation, whilst an inert (anaerobic) atmosphere would promote the utilisation of photoexcited electrons towards H^+^ reduction (as no O_2_ is present to compete for the electrons).^13,51^ The following sections discuss the obtained results starting from H_2_ generation rates of neat P25 and Pt-loaded P25, continuing with the discussion of CH_4_ production.

### Hydrogen production

**Fig. 2:**
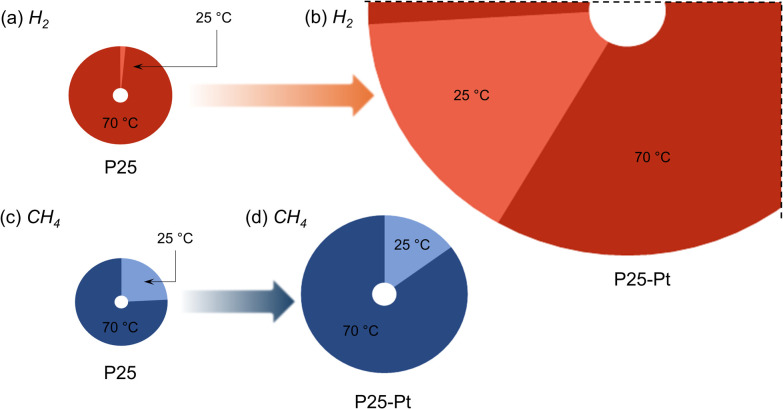
Gas phase analysis: schematic illustration of the relative amounts of solar fuels generated as a function of the catalyst and temperature depicting (a) H_2_ from P25, (b) H_2_ from P25-Pt, (c) CH_4_ from P25 and (d) CH_4_ from P25-Pt. Production values in µmol h^−1^ are presented in Table S2. Alternative bar graph view is shown in Fig. S6.

We observed quite low H_2_ production rates of 0.001 µmol h^−1^ when photoreforming PET using neat P25. The use of an elevated temperature (70 °C) boosted the hydrogen evolution reaction (HER) significantly, by a factor of ∼63 (Fig. 2a and Fig. S6a), leading to 0.071 µmol h^−1^ of H_2_ generated. Interestingly, in our reference experiments, we observed similar values for the HER and a similar effect of temperature when no PET was present in the reaction medium (Table S2). This result suggests that in the absence of a suitable co-catalyst, H_2_ evolution is not affected by the presence of PET and the prime source for H_2_ originates from the overall water splitting reaction. This is possible as the use of basic pH during the photoreforming favours water oxidation which often becomes the bottleneck of water splitting. We note, however, that the process runs with an unpractically low AQY of 0.00003% (Table S3).

As schematically shown in Fig. 2b, the presence of Pt on the TiO_2_ surface boosted the HER of neat TiO_2_ by 4 orders of magnitude, resulting in 2.87 µmol h^−1^ of H_2_ generated (AQY of 0.08%). This high H_2_ amount corresponds to approximately 20% of the PET being up-converted (Table S11 and SI Note 7) and indicates a very important role of Pt in realising efficient conversion of H^+^ into H_2_. This role is likely dual: it provides suitable proton adsorption and catalytic sites as well as strongly facilitates charge extraction, resulting in more efficient charge utilisation. Analysis of liquid-phase photoreforming products that will be discussed later further emphasises this favourable effect of Pt on the efficiency of hole utilisation, as it appears to promote the generation of AA from EG. For the P25-Pt photosystem (at RT), only a minor part of the generated H_2_ (∼40%) is produced in the absence of PET, suggesting that PET is essential for this high HER rate (Table S2). Besides this, similar to the effect of temperature on the HER performance of neat P25, we observed a further substantial increase of the H_2_ generation rate of P25-Pt at 70 °C, reaching as high as 15.35 µmol h^−1^ (Fig. 2b), corresponding to outstanding AQY values up to 0.45%.

### Methane production

Contrary to many other studies on PET photoreforming, we consistently observed generation of CH_4_ from the reaction mixture, on par with H_2_ production discussed above. CH_4_ is a high-value product with a 117.07 billion US$ ^52^ market share and a high price of €80 per MWh (similar to natural gas)^53^ compared to €30 per MWh for H_2_ from fossil fuels,^54^ with both automotive^52^ and energy sectors^55^ being highly interested in green CH_4_ sources. Photoreforming of PET using neat P25 yields a CH_4_ generation rate of 0.01 µmol h^−1^, which is 10 times higher than that of H_2_ production (Fig. 2c, Fig. S6b and Table S2). Increasing the temperature from RT to 70 °C further increases the CH_4_ yield 3-fold. When using the P25-Pt photosystem, similar to its increased performance towards the HER, we observed significantly more CH_4_ (3–7 fold increase, Fig. 2d) compared to neat P25. Our blank experiments performed in the absence of PET confirm that the majority of CH_4_ (up to 91%) originates from PET, while only a minor CH_4_ production in the absence of PET (as the only intentional C source) could be traced to carbon impurities (SI Note 4).

Since only a few early works have reported CH_4_ evolution from PET photoreforming, the mechanism of CH_4_ formation has not been investigated so far. Our datasets allow discussing possible pathways of CH_4_ generation, including (i) hydrogenation of C_1_ products of PET oxidation,^56^*e.g.*, *via* CO/CO_2_/HCO_3_^−^/CO_3_^2−^ reduction by molecular H_2_,^57–59^ or (ii) direct CH_4_ formation as a side-product of PET photodegradation, *e.g.*, *via* coupling of CH_3_ and H radicals. Regarding the first point: systematic analyses of both H_2_ and CH_4_ generation rates (Fig. 2 shows relative values, whereas Table S2 shows absolute values) reveal a strong but non-linear correlation between H_2_/CH_4_ production ratio change (increase or drop) as a factor of system parameters. As such, in RT photoreforming experiments, loading of P25 with Pt results in a significant increase in both the HER (∼1000 times) and CH_4_ production (∼4 times). In a similar manner, increasing the temperature from RT to 70 °C yields more H_2_ (∼200 times) and more CH_4_ (∼7 times). This disproportional increase of H_2_ and CH_4_ rates suggests that H_2_ production is only poorly correlated with CH_4_ generation, indicating that hydrogenation – path (i) – may not be the main mechanism of CH_4_ formation. A similar conclusion can be reached when analysing relative H_2_/CH_4_ generation ratios in P25 samples with and without Pt. In the absence of Pt, H_2_ and CH_4_ formation rates are on a similar level (size of the circles presented in Fig. 2a and c). When Pt is present, we see outstanding H_2_ evolution; however, the presence of such large amounts of H_2_ in the solution has a rather negligible effect on CH_4_ production (Fig. 2b and d). To further validate this result, we conducted photoreforming studies using P25-Pt (both at RT and 70 °C) under a CO_2_/H_2_ saturated atmosphere. Despite the availability of both H_2_ and CO_2_, we observed only a marginal effect on CH_4_ generation (SI Note 5), which corroborates that the hydrogenation pathway (i) plays a minor role in CH_4_ formation and rather points to the (ii) mechanism.

### Liquid-phase products

**Fig. 3:**
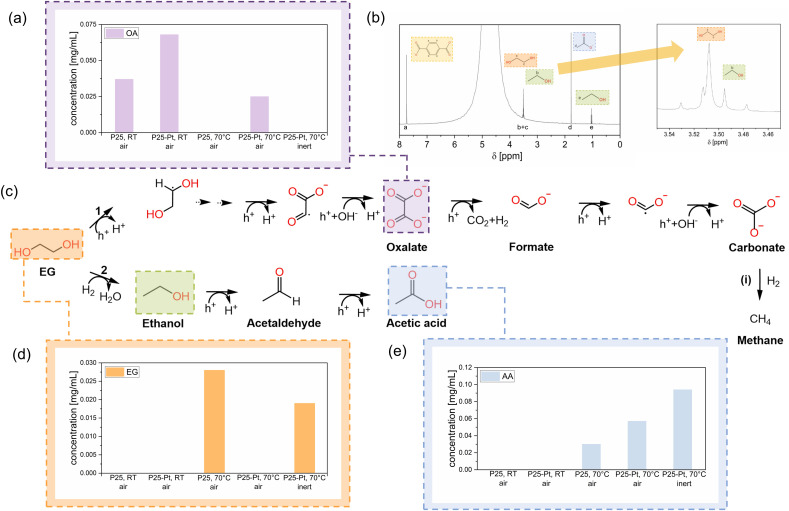
Liquid phase analysis: (a) HPLC analysis of OA, (b) ^1^H NMR spectrum of P25-Pt at 70 °C (inert, under a UV lamp), measured in D_2_O and 1 M NaOH (1 : 5), without water suppression, (c) suggested pathways of EG conversion into the detected products: (1) OA pathway and (2) AA pathway, including HPLC results from samples obtained under air and under inert conditions, and (d and e) obtained yields of EG and AA *via* HPLC analysis.

While the generation of solar fuels, H_2_ and CH_4_, constitutes an important milestone in the photoreforming process, elucidation of the liquid phase products provides complementary mechanistic insights and can further reveal the potential of the process to generate high-value compounds. The formation of PET photoreforming products in liquid phase was thus followed by high pressure liquid chromatography (HPLC) (Fig. 3a, d and e and Table S8) analysis and nuclear magnetic resonance (NMR) measurements (Fig. 3b). In addition, the accumulation of gaseous CO_2_ production in the reactor headspace was quantified by means of GC; however, we note that at high pH levels, CO_2_ is predominantly present in its mineralised forms as HCO_3_^−^ and CO_3_^2−^, which is in line with the low amounts of gaseous CO_2_ detected in our experiments^24^ (Table S9).

A key component of the PET photoreforming reaction is the alkaline media employed to facilitate the hydrolysis of PET to its monomers TP and EG. Whilst TP is generally regarded as inactive towards the following photocatalytic conversion,^60^ EG can be readily photoconverted, forming liquid products such as AA, FA, ethanol, OA, glyoxal and others (Fig. 3c).^19–21,23,61^ Our HPLC results indicate that a range of these compounds (along with unconverted TP) were found in our PET photoreforming experiments; however, the exact mixture composition depended strongly on the catalyst and conditions used. Interestingly, we observed that the temperature of the photoreforming has a strong effect on the relative EG accumulation (from PET hydrolysis) and consumption (from its photoconversion) rates. As such, EG was predominantly detected in the P25 sample at 70 °C, while it was more readily transformed into OA at RT (Fig. 3a). We also observed that higher temperatures generally facilitate the dehydroxylation pathway of EG (Fig. 3c, pathway 2). The presence of the Pt co-catalyst, on the other hand, only affected the mechanism of the EG conversion at 70 °C, which is reflected in the accumulation of OA under these conditions (Fig. 3a and c, pathway 1). In the absence of Pt, OA got converted into other (C_1_) compounds, with no detectable generation of FA for samples irradiated at 365 nm (Table S8), suggesting that OA gets fully oxidized to CO_2_/CO_3_^2−^ under these photoreforming conditions.^24^

Our results also show that the mechanism and product selectivity of PET photoreforming can be tuned by controlling the reaction atmosphere (Fig. 3a, d and e). As such, purging the solution of P25-Pt (reaction at 70 °C) with He resulted in approximately 1.5 times higher yield of AA compared to aerobic conditions. This is in line with the conclusions of Han *et al.*^23^ who suggested that less reductive conditions promote the dehydroxylation pathway (Fig. 3e and c, pathway 2) and convert EG predominantly into AA *via* ethanol and acetaldehyde. Our carbon balance calculations further indicate that around 30% of PET was successfully valorised to AA under these conditions, suggesting a very efficient photoreforming process (Table S10 and SI Note 6). The ^1^H NMR spectrum of the reaction solution (Fig. 3b) complements these findings: apart from the characteristic peak for aromatic protons of TP, a peak at around 1.8 ppm corresponding to acetate ions is observed along with a triplet at around 1.1 ppm, which may indicate the formation of ethanol. A zoomed in inset further reveals the characteristic CH_2_ quartet of ethanol (with *J* = 7 Hz) overlapping with the strong signal of the OH peak of EG at around 3.5 ppm. The absence of OA in the products obtained under an inert atmosphere (Fig. 3a) again suggests that it readily undergoes further oxidation. However, assuming that OA would eventually convert into CO_2_, it is surprising to see that P25-Pt produces much more CO_2_ (a factor of ∼6) when photoreforming is run at RT *vs.* 70 °C (Table S9). This can be linked to the fact that elevated temperature results in a much stronger CH_4_ production (refer to the discussion about solar fuels above) and suggests that CO_2_ can be consumed by its hydrogenation to some extent.

### Sunlight-driven photoreforming

The possibility of employing sunlight for PET photoreforming was explored by conducting experiments using solar-simulated light (details in Experimental and SI Note 8). The formation of both gas phase (Table S14) and liquid phase products (Fig. S9 and Table S8) was followed. In contrast to our results obtained under UV light, photoreforming of PET under solar light using neat P25 at 70 °C yielded only negligible amounts of H_2_ (a factor of ∼300 lower) and CH_4_ (a factor of ∼15 lower). Considering that both HPLC (Table S8) and NMR (Fig. S9) analyses only showed the monomers TP and EG and a negligible quantity of FA in the product mixture, we can effectively conclude that the photoreforming was largely unsuccessful under these conditions, presumably due to low conversion rates and insufficient intensity of incoming light. In contrast, solar-driven photoreforming of PET using P25-Pt at RT resulted in much stronger generation of both solar fuels, yielding 0.209 µmol h^−1^ of H_2_ and 0.009 µmol h^−1^ CH_4_. These rates are lower compared to those obtained under a UV LED source (a factor of ∼14 for H_2_ and ∼4.5 for CH_4_ compared to Table S2); however, considering the low proportion of UV photons in the solar spectrum, one arrives at a UV-based AQY value of 0.84% for P25-Pt based on the H_2_ generation rates. As expected, P25-Pt also produced a number of liquid phase compounds with a composition similar to that obtained under UV light. These results and respectable AQY obtained for P25-Pt under solar-simulated light highlight the important role of Pt in solar-driven PET photoreforming and further showcase the commercial potential of the process (SI Note 8).

### Degradation pathways and long-term performance

**Fig. 4:**
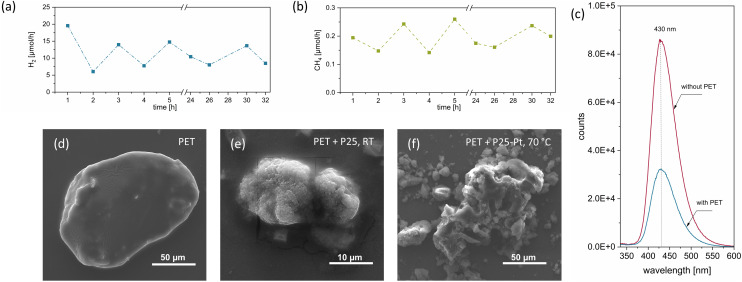
Mechanism, long-term stability and microplastic degradation: generated H_2_ (a) and CH_4_ (b) in the course of 32 h (1 mg mL^−1^ PET, 0.5 mg mL^−1^ P25-Pt (1 wt%), 100 mL of 1 M NaOH, RT); (c) PL emission spectrum of P25-Pt (1 wt%) with and without PET, under He and at RT; SEM images of (d) unmodified PET, (e) PET particles after irradiation with P25 (at RT), and (f) after irradiation with P25-Pt (at 70 °C).

SEM images of the neat and photoreformed PET powders are shown in Fig. 4. The unmodified PET (Fig. 4d) depicts a smooth surface with well-defined edges characteristic of soft matter, whereas micrographs of PET particles after reaction (Fig. 4e and f) illustrate a less distinct structure and reveal sharp edges indicating that some form of decay and physical degradation has taken place upon irradiation. SEM images also indicate that a close contact between TiO_2_ particles and PET particles is established during the photoreforming process, although zeta potential measurements (Fig. S11 and Table S17) revealed that both the powders (photocatalyst and microplastic) exposed to alkali medium show negative charge values well below −20 mV. This suggests that van der Waals attraction between PET and TiO_2_ still dominates in the solution,^62^ leading to the close interface between the two. Photoluminescence spectroscopy studies employing ˙OH scavengers were conducted to elaborate on the contributions of indirect and direct oxidation pathways towards the PET degradation process with P25-Pt (details in SI Note 10).^20,63,64^ The results in Fig. 4c indicate that the generation of ˙OH is impeded in the presence of PET, thus confirming that direct hole transfer through the TiO_2_/PET interface established in the reaction suspension plays an important role in the process of PET particle activation and conversion. Besides this, we also conducted photocatalytic experiments in the presence of radical quenchers (SI Note 10) capable of selectively trapping various reactive oxygen species. Fig. S13 shows that only benzoquinone resulted in a strong activity drop (99.7% less H_2_ and 94.3% less CH_4_ was produced in its presence), which hints to the importance of superoxide radicals in the process of PET oxidation.

Long-term performance stability was next evaluated over the course of 32 hours of irradiation and *via* a large-scale implementation by around 5-fold (SI Note 11). Fig. 4a and b show the rates of H_2_ and CH_4_ generation as a function of time. While we observed strong hour-to-hour fluctuations in activity, which might be related to bubble evolution dynamics, an overall stable generation of both solar fuels can be concluded. Selected catalytic suspensions were further recovered by filtration and analysed using XRD. The obtained XRD pattern of the recovered solid (Fig. S14) shows the characteristic peaks of rutile and anatase phases of P25 (suggesting the catalyst's stability), along with additional peaks corresponding to unprocessed PET. No morphological or phase change has been observed, which is in line with the stable catalytic performance of the P25-Pt photocatalyst.

## Conclusions

In this study, we explored the potential of P25-based photosystems towards PET photoreforming and highlighted several outcomes that are of interest for further development of the process. We found that photoreforming PET using neat P25 results in marginal H_2_ production, which can be increased by 1–2 orders of magnitude by elevating the reaction temperature. In contrast to this, the addition of Pt significantly boosted hydrogen evolution of P25 by 4 orders of magnitude, which highlights the crucial role of Pt in promoting the reaction of interest. A mixed use of Pt and elevated temperatures yielded synergistic HER improvement, resulting in more than 15 µmol h^−1^ of H_2_ corresponding to AQY values up to 0.45%. Besides detecting H_2_, we observed significant CH_4_ production during PET photoreforming, with rates exceeding H_2_ generation using neat P25 and further increasing with temperature and Pt addition. Mechanistic investigations suggest that CH_4_ primarily originates from PET degradation rather than hydrogenation of PET oxidation products, as CH_4_ production remains largely independent of H_2_ concentration. Liquid phase analysis revealed that reaction conditions and the catalyst type significantly influence product distribution, with EG photoconversion leading to various intermediates such as AA, OA, FA and ethanol. Some product selectivity was observed, with high AA yields further enhanced by the presence of Pt and OA production preferred under aerobic conditions. Furthermore, the possibility of large-scale implementation was demonstrated through irradiation in a 100 mL setup for 32 hours and experiments simulating the solar spectrum for 24 hours. The promising AQY values indicate the potential of this technology, as P25-Pt yielded significant amounts of both solar fuels and remained stable for the prolonged irradiation period in the larger setup. It is clear that PET not only acts as a sacrificial agent for the formation of H_2_ but also shows great potential for the generation of valuable chemicals and green CH_4_. While this investigation offers useful new insights for the development of large-scale application, further in-depth mechanistic studies are crucial for advancing this sustainable abatement method and realising the selective photoconversion process.

## Experimental

### Pt photodeposition on P25 TiO_2_

For the photodeposition of 1 wt% Pt on P25 TiO_2_, 1 g of the support was transferred to a round bottom flask containing 200 mL of a 1 : 1 MeOH : H_2_O solution. Then 12.5 mL of a 20 mmol H_2_PtCl_6_ precursor solution was added. The suspension was purged with Ar for 5 minutes, before starting double sided illumination (top and side) with two UV light sources (365 nm SOLIS LED and Lumatec LED lamps), with wavelengths in the range of 190–400 nm for 135 minutes. Subsequently, the MeOH : H_2_O mixture was removed and the photocatalyst was dried overnight at 60 °C.

### Photoreforming experiments

In a standard run, the generation of solar fuels was investigated by adding 18 mg of PET powder (1.0 mg mL^−1^) and 9 mg (0.5 mg mL^−1^) of the respective photocatalyst (either neat P25 or P25-Pt) to a 20 mL vial with a septum, containing 18 mL of 1 M NaOH solution. The suspension was sonicated for a total of 12 minutes to achieve good dispersion of the powders. The employed setup did not include a water cooling system and increased temperature was achieved with a predetermined setting of the hot plate. Before irradiating the respective sample, the solution was purged with either helium or air for 10 minutes – closing the system to achieve inert conditions or continuing purging with air for the entire duration of the experiment. Illumination was performed with a 365 nm SOLIS LED lamp, with a measured intensity of 69.7 mW cm^−2^, for 5 hours under magnetic stirring. The generated quantities of both H_2_ and CH_4_ were detected by GC. The liquids and solids were separated *via* filtration and kept for further analysis.

### Solar-simulated photoreforming experiments

The general procedure of these two samples is identical to the aforementioned description, with the exception of decreasing the suspension from 18 mL to 16 mL and thus altering the mass of PET and the photocatalyst to use the same concentrations as used in our standard photoreforming studies. Irradiation was performed for 24 hours, implementing a broad band Xe lamp with an IR filter and an intensity of 21.3 mW cm^−2^.

### Scale-up experiment

Herein, the suspension volume was increased to 100 mL and irradiation was performed in a round bottom flask equipped with a septum. The suspension was sonicated for 17 minutes and purged with He for 30 minutes. The concentration of PET and P25-Pt remained at 1.0 mg ml^−1^ and 0.5 mg mL^−1^, respectively. The sample was illuminated under magnetic stirring at 365 nm (SOLIS LED) with an intensity of 69.7 mW cm^−2^ for 32 hours, extracting and measuring gaseous samples every hour within the first 5 hours of the experiment and then every 2 hours between the 24 and 32 hours, to investigate the activity and long-term performance of the photocatalytic system. The solution was separated from the remaining solids and stored for further characterisation.

## Author contributions

Conceptualisation: MW and AC; methodology: MW and AC; investigation: MW, AE, CvB, and AK; resources: CP, FK, DE, and AC; data curation: MW; writing—original draft preparation: MW and AC; writing—review and editing: MW, AE, CvB, AK, CP, FK, DE and AC; visualisation: MW; supervision: AC; project administration: AC; funding acquisition: AC. All authors have read and agreed to the published version of the manuscript.

## Conflicts of interest

There are no conflicts to declare.

## Supplementary Material

GC-028-D5GC03562G-s001

## Data Availability

The data supporting the findings of this study, including characterization results (microscopy, XRD, NMR, UV-vis DRS, PL, GC, HPLC, TXRF, zeta-potential), and supplementary analysis protocols (*e.g.*, AQY calculations), are provided in supplementary information (SI). See DOI: https://doi.org/10.1039/d5gc03562g. The raw research data supporting the main text figures have been deposited in TU Wien Research Data Repository and are openly available at https://researchdata.tuwien.ac.at/records/k185m-c6a84. Any additional information will be provided upon request.
