# Supplementary material for: Photocatalytic upcycling of PET into methane, hydrogen and high-value liquid products
Source: Green Chem. 2025 Nov 12;28(1):309–17. doi: 10.1039/d5gc03562g (PMC12645822; doi:10.1039/d5gc03562g)
Supplement: GC-028-D5GC03562G-s001 [file GC-028-D5GC03562G-s001.pdf]

Supplementary Information (SI) for Green Chemistry.
This journal is © The Royal Society of Chemistry 2025

Supplementary Information

Photocatalytic Upcycling of PET into Methane, Hydrogen and High-value Liquid Products

Madeline Weisweiller[a], Adrian Ertl[a], Cornelia von Baeckmann[a], Anil Kumar Sihag[b,c], Christian M. Pichler[b,c], Freddy Kleitz[d], Dominik Eder[a] and Alexey Cherevan[a]*

[a] *Institute of Materials Chemistry, TU Wien, Getreidemarkt 9/BC/02, 1060, Vienna, Austria*

[b] *CEST - Centre for Electrochemistry and Surface Technology, Wr. Neustadt, Viktor-Kaplan-Straße 2 2700 Wiener Neustadt, Austria*

c Institute of Applied Physics, TU Wien, Wiedner Hauptstraße 8, 1040, Vienna, Austria

[d] *Department of Functional Materials and Catalysis, Faculty of Chemistry, University of Vienna, Währinger Straße 42, 1090 Vienna, Austria*

Table of Contents

List of chemicals ... *3*
Characterisation methods ... *4*
Gas chromatography (GC) ... 5
Apparent quantum yield (AQY) calculations ... 6
High pressure liquid chromatography (HPLC) ... 4
Scanning electron microscopy (SEM) ... 4
Transmission electron microscopy (TEM) and Energy dispersive X-ray analysis (EDX) ... 4
Powder X-ray diffraction (XRD) ... 4
Nuclear magnetic resonance spectroscopy (NMR) ... 4
UV-vis Diffuse reflectance spectroscopy (DRS) ... 4
Total reflection X-ray fluorescence (TXRF) ... 5
Zeta-potential measurements ... 5
Photoluminescence spectroscopy (PL) ... 5
Supplementary note 1. Material characterisation ... *7*
Supplementary note 2. Workflow of the photoreforming studies ... *9*
Supplementary note 3. Analysis of blank samples ... *10*
Supplementary note 4. Origin of methane ... *11*
Supplementary note 5. Methane formation pathways ... *12*
Supplementary note 6. Liquid phase analysis ... *13*
Supplementary note 7. PET conversion to gas phase products ... *15*
Supplementary note 8. Solar-light-driven photoreforming ... *17*
Supplementary note 9. Window sill experiments ... *19*
Supplementary note 10. PET degradation mechanism ... *20*
Supplementary note 11. Upscaled experiment ... *24*
Supplementary note 12. Images of the reaction solutions ... *25*
References ... *27*

List of chemicals

All compounds utilised throughout this work were commercially obtained and used without further modifications.

Chemical formula	Name	CAS number	Purity	Supplier
TiO_2	Titanium(IV)oxide, P25	13463-67-7	-	Acros Organics
H_2PtCl_6	Hydrogen hexachloroplatinate (IV)	16941-12-1	-	Fluka (Sigma – Aldrich)
CH_3OH	Methanol	67-56-1	Absolute (≥99,9%)	VWR
NaOH	Sodium hydroxide	1310-73-2	≥99,0%	Carl Roth
$(C_{10}H_8O_4)_n$	Polyethylene Terephthalate	26038-69-9	99,0%	Nanochemazone
$C_2H_6O_2$	Ethylene glycol	107-21-1	99,5%	Acros Organics
$C_2H_2O_4 * 2H_2O$	Oxalic acid dihydrate	6153-56-6	99,0%	Sigma-Aldrich
CH_3COOH	Acetic acid	7732-18-5	98-100%	Fisher Scientific
H_3PO_4	Ortho-phosphoric acid	7664-38-2	85,0%	PanReac AppliChem - ITW reagents
$C_8H_6O_4$	Terephthalic acid	100-21-0	For synthesis	Sigma Aldrich

Characterisation methods

A variety of techniques were carried out to investigate the prepared photocatalysts and the formed gaseous and liquid products from the photoreforming process. A brief description of each applied method is provided below.

High pressure liquid chromatography (HPLC)

For the determination and quantification of oxidation products, the following standard solutions were prepared with deionised water: ethylene glycol, acetic acid, oxalic acid and formic acid. The calibration curves of the respective compounds were gathered from solutions of 10.0 mg/mL, 1.0 mg/mL and 0.1 mg/mL concentration, respectively. Each sample under investigation was neutralised with H_3PO_4 to pH 2-7 and then filtered with a syringe filter for subsequent analysis. All liquid products were analysed by HPLC using a Shimadzu LCMS-2020 liquid chromatograph – mass spectrometer. The analytes were separated with a Shim-pack SCR-102H ion-exchange column (300 × 8.0 mm, 7 µm) maintained at 70 °C. Detection was performed using a refractive index detector (Shimadzu RID-20A). A 10 µL analyte volume was injected, with 0.1 wt% aqueous H_3PO_4 as the mobile phase, maintained at a constant flow rate of 0.8 mL/min.

Scanning electron microscopy (SEM)

To evaluate the change of morphology upon photoreforming, SEM images were attained with a FEI Quanta 250 FEG scanning electron microscope. An acceleration voltage of 20 kV and secondary electron detection mode were generally used.

Transmission electron microscopy (TEM) and Energy dispersive X-ray analysis (EDX)

TEM images of the prepared P25-Pt photocatalyst were attained at an operating voltage of 200 kV, employing a Tecnai F20 FEG transmission electron microscope configured with a Gatan Rio16 CCD-camera and an EDAX-AMETEK Apollo XLTW SDD EDX-detector.

Powder X-ray diffraction (XRD)

The photocatalyst and some samples obtained after irradiation (including the substrate), were investigated via powder XRD, to examine possible changes in crystallinity and phase composition of the respective sample. Herein, the XPERT II: PANalytical XPert Pro MPD ((θ–θ Diffractometer, PANalytical, Almelo, The Netherlands) was employed for analysis. For each measurement, irradiation was carried out with a Cu X-ray source (8.05 keV, 1.5406 Å), attaining data from 5 to 90 degrees, applying Bragg–Brentano θ–θ -diffractometer geometry and using a semiconductor X'Celerator (2.1°) as detector. The desired parameters were then evaluated by implementing Rietveld refinement with the software "Highscore" and by employing the ICSD (inorganic crystal structure database) database for peak assignment.

Nuclear magnetic resonance spectroscopy (NMR)

NMR measurements were carried out for the obtained liquid phases using a Bruker Bruker AVANCE 250 (250.13 MHz) with a z-gradient unit and 5 mm inverse-broad probe head. For each analyte, D_2O was added to the reaction solutions containing 1M NaOH, in a 1:10 or 1:5 ratio. Measurements were carried out with and without water suppression.

UV-vis Diffuse reflectance spectroscopy (DRS)

The evaluation of absorption characteristics of respective solid powder samples was carried out employing the Jasco 670 UV-Vis-NIR spectrophotometer (Tokyo, Japan). Herein, the reflectance spectra were attained at wavelengths from 200-900 nm and using a Teflon block as reference. Acquired data was then converted from reflectance (R) to absorbance (A), via *Eq. 3*.

$$A\,(a.u.) = \,log_{10}\frac{1}{R} \qquad \textit{(Eq. 3)}$$

Total reflection X-ray fluorescence (TXRF)

After co-catalyst deposition, the exact loading of Pt (in wt%) was evaluated via TXRF measurement. Herein, an ATOMIKA 8030C X-ray fluorescence analyser was employed, equipped with a Mo X-ray tube to excite the sample (using monochromatized Kα-line) at 50 kV and 47 mA for 100 seconds. Further, an energy-dispersive Si(Li)-detector and total reflection geometry to enhance sensitivity were implemented. Minor amounts of the sample were placed on a quartz reflector using a pipette tip, followed by the addition of 5 µL of a 1% polyvinyl alcohol solution (PVA). The sample was then dried on a hot plate at approximately 100 °C for 5 minutes to ensure adhesion of the powder to the reflector. Before measurement, blank reflectors were analysed to rule out the presence of contaminants. To elucidate the wt% of Pt, Ti was set as the matrix at 100% and *Eq. 4* was applied. *(M(Ti) = 47.87 g/mol, M(TiO_2) = 79.87 g/mol, measured c(Pt) = 1.666 ppm)*

$$\frac{M(Ti)}{M(TiO_2)} * c(Pt) = m(Pt) \text{ in wt\%} \qquad \textit{(Eq. 4)}$$

Zeta-potential measurements

Zeta-potential measurements were performed using a Malvern Zetasizer Nano ZS. Stable aqueous suspensions of the material in water or 0.01 M NaOH (1 - 0.5 mg/mL) were obtained by performing cycles of vortex shaking and ultrasonic bath treatment. pH adjustment was performed using 0.2 M NaOH and 0.5, 0.1 and 0.01 M HCl solutions. The suspension was analysed to obtain zeta-potential values. Measurements were accepted as valid only when satisfactory quality criteria such as good distribution data and count rate were fulfilled. Prior to zeta-potential measurements, a standard solution with a zeta-potential of −42 ± 6 mV was measured to ensure correct calibration. Zeta-Potentials and are reported as an average of six measurements, with each measurement consisting of 20-200 runs.

Photoluminescence spectroscopy (PL)

To elucidate on the photocatalytic mechanism, PL was carried out using a FluoTime 300 spectrometer. Samples and blanks were prepared by simulating reaction conditions with 0.003 M terephthalic acid (prepared with 1M NaOH) as reaction medium instead of pure 1 M NaOH. After 5 hours of irradiation, the solutions were filtered and then employed for steady state measurements. Herein a Coaxial UV-Xenon arc lamp was implemented with a high-resolution excitation double monochromator, exciting the samples at 315 nm. Emitted photons were quantified with a PMA Hybrid 07 detector, equipped with a high-resolution emission double monochromator, with formed 2-hydroxyterephthalic acid showing an emission maximum around 430 nm. Moreover, steady state PL measurements were carried out with the bare P25 powder and P25-Pt employing the FluoTime 300 spectrometer (excitation at 378 nm) to investigate the effect of the Pt co-catalyst on charge separation.

Gas chromatography (GC)

The gaseous product generation of H_2 and CH_4 was studied via GC (Shimadzu Nexis GC-2030 BID). Received data was extracted from 200 µL samples from the headspace, measuring after irradiation. The calculated ppm values from the software were then converted to µmol and µmol/h, applying the following equations (*Eq. 1* and *Eq.2*). *(p = 101325 Pa; R = 8,314 m^3Pa/molK; T = 298K for 25 °C or 343K for 70 °C)*

$$p * V = n * R * T \rightarrow n = \frac{p*V}{R*T} \qquad \textit{(Eq. 1)}$$

$$\mu mol = \frac{concentration\,[ppm] * headspace\,[mL] * 10^{-6} * p\,[Pa]}{R\left[\frac{m^3 * Pa}{mol * K}\right] * T\,[K]} \quad (Eq.2)$$

Apparent quantum yield (AQY) calculations

The AQY is defined as the ratio of number of electrons that have reacted (generating H_2) to the number of photons absorbed. The light intensity was assessed by employing a PM100D power meter from Thorlabs (67.13 mW/cm^2 for UV light source, 25.48 mW/cm^2 for Xe lamp). Moreover, the number of photons absorbed by the reaction solution (16 or 18 mL 1M NaOH + 1 mg/mL PET powder + 0.5 mg/mL P25-Pt) was determined by positioning the power meter directly behind the illuminated vial and obtaining values with and without the vial at the position. The difference between these two values demonstrate the amount of light absorbed by P25-Pt.[1,2] For the Xe lamp, the measured intensity values were multiplied with 4%, representing the UV portion of the solar spectrum.[3] In addition, when assessing AQY values of the experiments conducted using simulated solar light, an average wavelength of 315 nm was selected (within a range of 280-380 nm), to facilitate the comparison between the two light sources.

Supplementary note 1. Material characterisation

To eliminate or at least dramatically reduce any carbon contaminants from the commercially obtained P25 TiO_2 powder, the semiconductor was calcined at 500 °C for 3 hours (the use of this sample as a reference is described in Supplementary note 4). The resulting powder was utilised for Pt photodeposition, as described in Methods section. XRD analysis of the commercial and pre-calcined P25 TiO_2 powder (Figure S1a) shows the characteristic peaks for the anatase and rutile phase. As suggested in literature[4] a phase transition of anatase to rutile can be initiated from calcination at 500 °C. The commercial P25 shows a composition of 10.7% rutile to 89.3% anatase. Conversely, the calcined sample illustrates only a minor (0.6%) increase in the rutile phase suggesting that no strong effect on the photocatalytic performance shall be expected from the rutile-anatase ratio change. DRS analyses (Figure S1b) similarly show only negligible change in absorption profile between the powders before and after calcination. Additionally, steady-state PL measurements (Figure S2) of both the neat photocatalyst and P25-Pt were performed at 378 nm, revealing a decrease in emission intensity upon the introduction of the noble metal. This reduction is attributed to the diminished recombination processes resulting from charge extraction by the Pt nanoparticles.

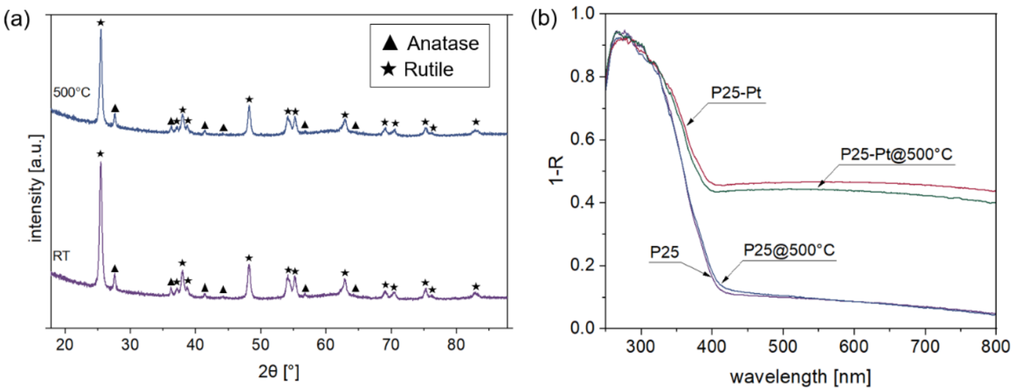

Figure S1: (a) XRD pattern of commercially obtained P25(RT) and P25 calcined at 500 °C) and (b) DRS measurement of neat P25 and with 1 wt% Pt nanoparticles compared to the calcined analogues.

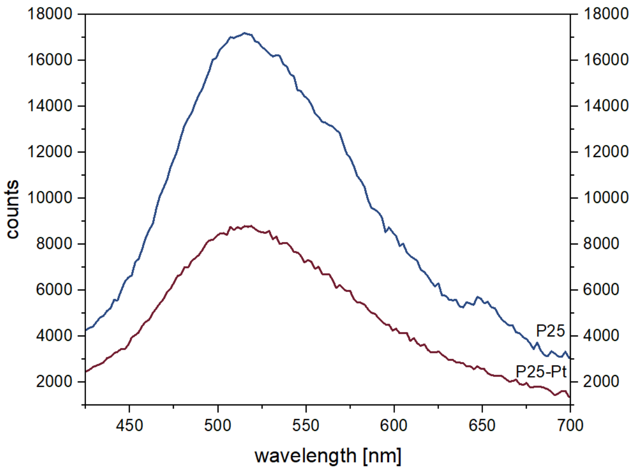

Figure S2: Steady state PL measurement of P25 and P25-Pt at 378 nm.

Furthermore, no morphological variation of P25 powder after calcination at 500 °C can be distinguished from the SEM images (Figure S3). Porous nature of the aggregates is evident from the images as spherically shaped TiO_2 particles clustered together, resulting in a sponge-like structure.

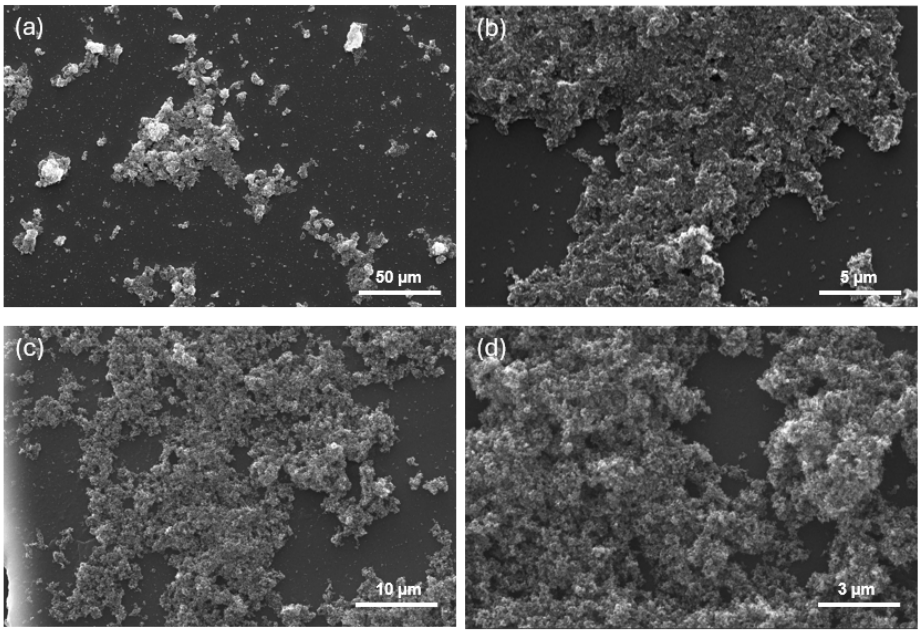

Figure S3: (a)and (b) unmodified P25, (c) and (d) P25 after calcination at 500 °C for 3h.

In the course of TEM measurements, EDX analysis was performed for one of the areas of the P25-Pt powder (Figure S4a) to determine the elemental distribution of O, Ti and Pt (Figure S4b). An average value of theoretically calculated values of the sum spectrum, unallocated phase and TiK/O K phase is presented in Table S1.

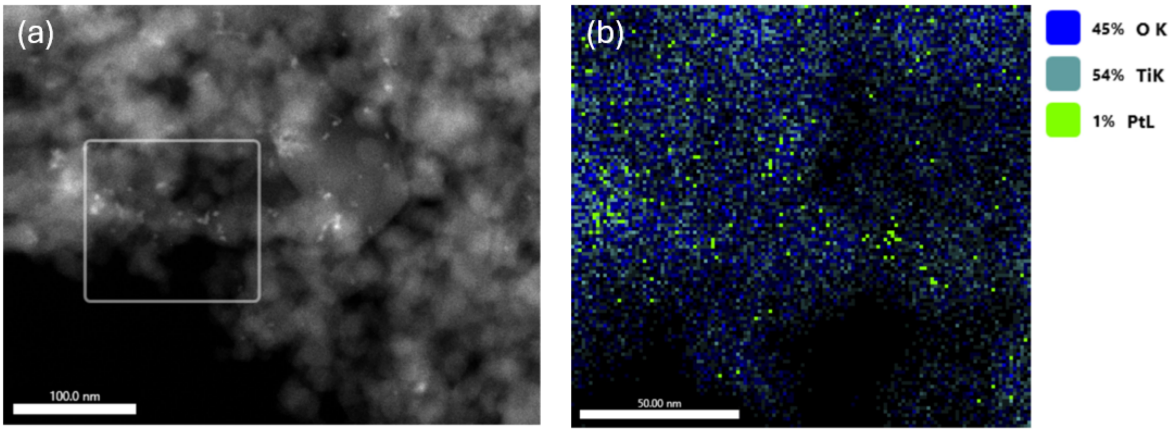

Figure S4: Image (a) and elemental distribution (b) of the P25-Pt photocatalyst under EDX analysis.

Table S1: Average of theoretically attained quantity of Ti and Pt, given in wt% and at%.

Element	wt%	at%
Ti	51.2	26.3
Pt	1.1	0.2

Supplementary note 2. Workflow of the photoreforming studies

In a typical experiment (either carried out in a 20 mL vial with septum or in a round bottom flask (upscaled experiment)) the reaction solution containing 1 M NaOH, PET and the catalyst was irradiated for 5h/24h/32h with a 365 nm LED or broad band Xe light source. Generated solar fuels in the headspace were analysed after different time intervals via GC by probing the reactor headspace with a gas tight syringe. Liquid phase analysis took place after the reaction was complete and the solid was filtrated by means of NMR and HPLC measurements. The process is schematically shown in Figure S5.

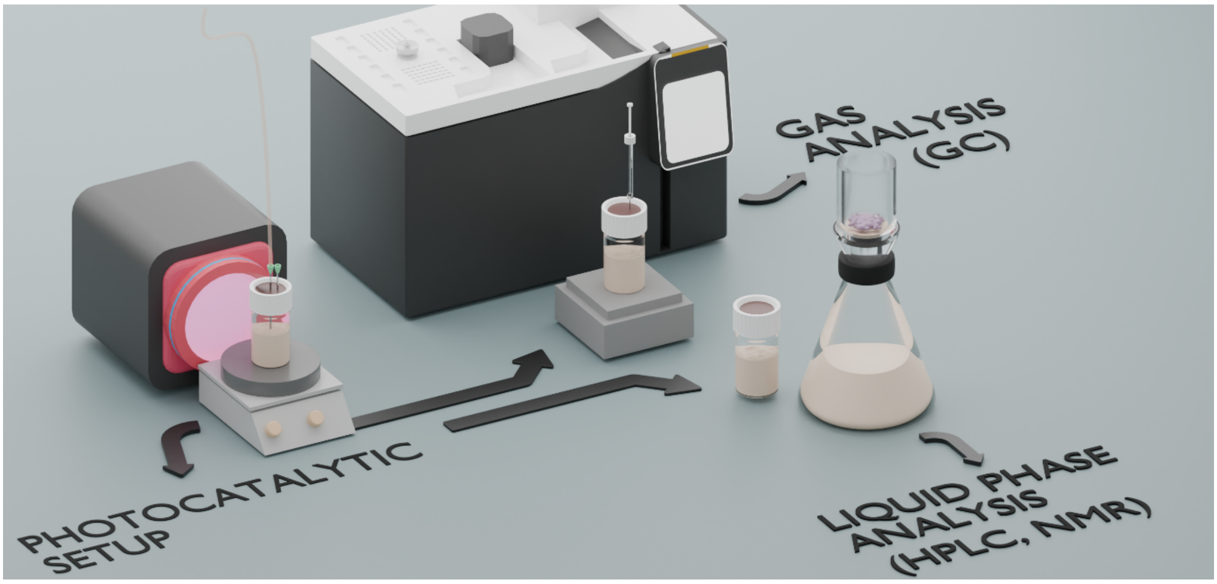

Figure S5: Schematic illustration of the experimental protocol followed to conduct photoreforming studies.

Supplementary note 3. Analysis of blank samples

During this study, a comprehensive set of blank and reference experiments were conducted to elucidate the possible reaction pathways towards H_2 and CH_4. The results are depicted in Table S2, Table S3 and Figure S6 below.

Table S2: Generated H_2 and CH_4 values from the respective catalysts at given temperatures, comparing scenarios where PET was and was not present in the reaction suspension.

sample	With PET		Without PET	
	H_2 [µmol/h]	CH_4 [µmol/h]	H_2 [µmol/h]	CH_4 [µmol/h]
P25-Pt/RT	2.870	0.042	1.250	0.006
P25/RT	0.001	0.011	0.008	0.002
P25-Pt/70 °C	15.35	0.243	0.998	0.035
P25/70 °C	0.071	0.035	0.519	0.022

Table S3: Calculated AQY values in % from the H_2 values of the samples summarised in Table S2.

sample	With PET	Without PET
	AQY [%]	AQY [%]
P25-Pt/RT	0.0838	0.0364
P25/RT	0.00003	0.00027
P25-Pt/70 °C	0.4488	0.0292
P25/70 °C	0.0021	0.0152

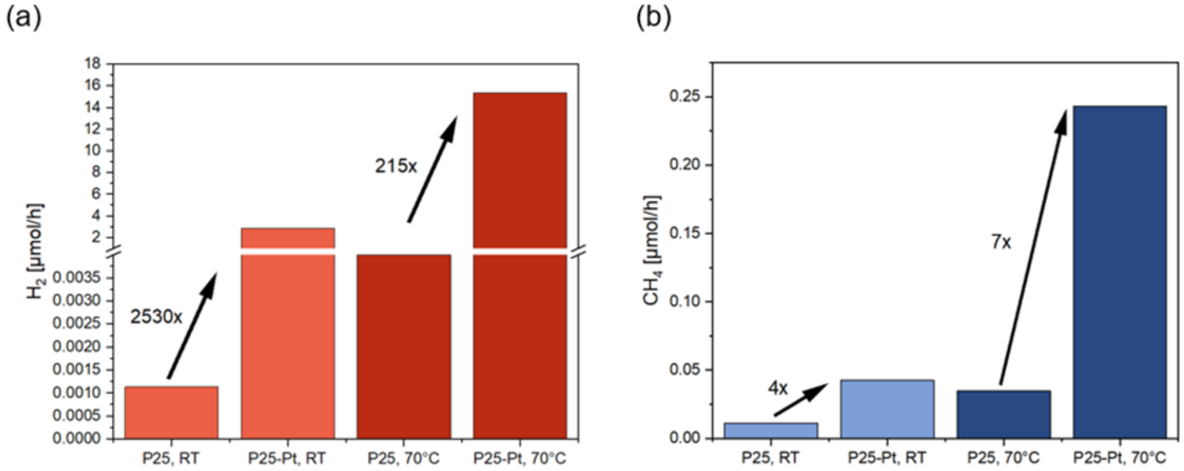

Figure S6: H_2 and CH_4 yields, as summarised in Table S2 and schematically illustrated in Fig. 2.

Supplementary note 4. Origin of methane

P25 TiO_2 is a highly dispersed, commercial-grade material produced by Evonik (formerly Degussa) using a specialized flame hydrolysis process of $TiCl_4$. Despite the production is solvent-free, the purity of the resulting TiO_2 is around 99.5% and the resulting product contains a small amount of carbon impurities. One type of carbon (bulk) is related to the trace environmental contamination during the complex industrial process, the other (surface) is also unavoidable and relates to carbon-containing species from the atmosphere (like CO_2 or VOCs) that adsorb onto the high-surface-area particles after manufacturing, during handling and storage. It is this surface contamination (often referred to as adventitious carbon) that we believe is the source of small amount of CH_4 generated in our photoreforming experiments in absence of PET. It is also known that calcination at temperatures around 500 °C in air can be used to effectively clean the surface and remove adsorbed organic or carbonaceous species before use. Therefore, to elucidate on the origin of generated CH_4, commercially obtained P25 was calcined at 500 °C for 3 hours aiming to reduce the carbon impurities as a potential source for generated CH_4. Subsequently, the samples and blanks, summarised in Table S4, were prepared and analysed. Herein, the reaction conditions remained indifferent to the non-calcined samples. The percentual difference in the amounts of obtained products between the calcined samples containing PET powder in contrast to the blanks is illustrated in Table S5. The results indicate that the addition of PET increases the generation of both H_2 and CH_4 substantially, with increases of up to 99.7% and 90.7% respectively. Herein, an AQY of 0.31% was obtained (Table S6).

Table S4: Generated H_2 and CH_4 values from the respective catalysts calcined at 500 °C, comparing samples with and without PET present.

sample	With PET		Without PET	
	H_2 [µmol/h]	CH_4 [µmol/h]	H_2 [µmol/h]	CH_4 [µmol/h]
P25-Pt/RT	1.68	0.020	1.62	0.012
P25/RT	0.002	0.004	0.002	0.001
P25-Pt/70 °C	10.733	0.184	0.035	0.017
P25/70 °C	0.229	0.039	0.186	0.010

Table S5: Percentual difference between sample and respective blank of carried out experiments with photocatalysts calcined at 500 °C, in regard to quantity of generated solar fuels.

sample	H_2	CH_4
	Sample vs. Blank	Sample vs. Blank
P25-Pt/RT	+ 3.7%	+ 43.5%
P25/RT	+ 42.9%	+ 81.8%
P25-Pt/70 °C	+ 99.7%	+ 90.7%
P25/70 °C	+ 18.9%	+ 74.3%

Table S6: AQY values in % calculated from the H_2 values of the samples summarised in Table S4.

sample	With PET	Without PET
	AQY [%]	AQY [%]
P25-Pt/RT	0.0491	0.0473
P25/RT	0.0001	< 0.0001
P25-Pt/70 °C	0.3138	0.0010
P25/70 °C	0.0067	0.0054

Supplementary note 5. Methane formation pathways

The hydrogenation pathway for the formation of CH_4 was explored by carrying out experiments involving the injection of 1000 µL of a CO_2+H_2 gas mixture (25:75 vol%) into the headspace after purging with He (Figure S7 and Table S7). Despite the presence of excessive amounts of H_2 and CO_2 available for the assumed hydrogenation process at the beginning of the reaction, analysis of the reaction products after the photoreforming run (5 h following the standard protocol described in *Methods*) revealed only minor elevation of CH_4 amount by approximately 1.5-fold at RT compared to the standard case (He only). At the same time, a slight decrease in CH_4 production was observed at 70 °C. Our data also confirm that in both cases the entire CO_2 amount was successfully taken up by the alkali solution (thus no measurable CO_2 in the headspace after reaction), making CO_2 available for the potential reaction in the solution. These findings point to the disconfirmation of the hydrogenation mechanism and rather support the idea that CH_4 is primarily formed via radical coupling mechanisms involving species directly formed from the PET.

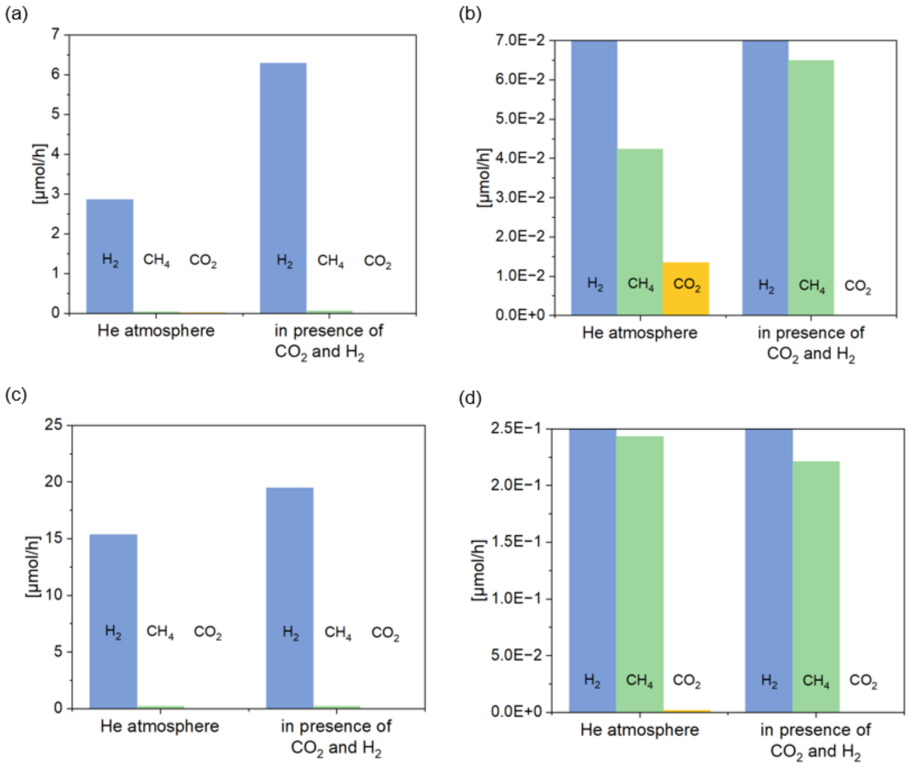

Figure S7: H_2, CH_4 and CO_2 yields from photoreforming of PET with P25-Pt at RT ((a) and (b)) and 70 °C ((c) and (d)), investigating the hydrogenation pathway for the formation of CH_4. (b) and (d) are magnified versions of (a) and (c), respectively.

Table S7: Comparing values of generated solar fuels from inert atmosphere (He) or considering the injection of 1000 µL of a CO_2 + H_2 gas mixture, shown in µmol/h, as illustrated in Figure S5.

Sample	H_2 [µmol/h]	CH_4 [µmol/h]	CO_2 [µmol/h]
P25-Pt/RT	2.87	0.042	0.014
P25-Pt/RT with CO_2 + H_2	6.28	0.065	0
P25-Pt/70 °C	15.35	0.243	0.002
P25-Pt/70 °C with CO_2 + H_2	19.47	0.221	0

Supplementary note 6. Liquid phase analysis

Experiments carried out under inert (purged with He) or aerobic (purged with air) conditions and irradiated for 5 hours with a 365 nm light source were investigated in respect to oxidation products in the liquid phase. Additionally, experiments were conducted with a round bottom flask with greater reaction volume ("upscaled") and with a broad band light source simulating solar spectrum ("Xe lamp"). Obtained results via HPLC analysis are summarised in Table S8. Moreover, measured CO_2 values of samples purged with He are illustrated in Table S9.

Table S8: Measured concentrations of ethylene glycol, oxalic acid, acetic acid and formic acid upon HPLC analysis.

Sample	Ethylene glycol [mg/mL]	Oxalic acid [mg/mL]	Acetic acid [mg/mL]	Formic acid [mg/mL]
P25/RT-air		0.037		
P25-Pt/RT-air		0.068		
P25/70 °C-air	0.028		0.030	
P25-Pt/70 °C-air		0.025	0.057	
P25-Pt/70 °C-inert	0.019		0.094	
P25-Pt/RT-inert/upscaled		0.005	0.081	
P25-Pt/RT-inert/Xe lamp	0.005		0.021	0.009
P25/70 °C-inert/Xe lamp	0.159			0.080

Table S9: Measured CO_2 rates for experiments purged with He, see samples discussed in Table S2.

Sample	CO_2 [µmol/h]
P25-Pt/RT	0.014
P25/RT	0.004
P25-Pt/70 °C	0.002
P25/70 °C	0.019

Moreover, the highest concentration of liquid phase product obtained from the photoconversion of ethylene glycol was further quantified. Hereby the sample comprised of P25-Pt and irradiated at 70 °C showed the most promising results, yielding 0.094mg/mL of acetic acid (Table S8). The equations below (*Eq. 6* and *Eq. 7*) describe the calculations of the values shown in Table S10. Hereby, it is assumed that per mol PET monomer unit, one mol acetic acid is produced via oxidation of the monomeric unit ethylene glycol, as shown in the reaction mechanism (*Eq. 5*). For the sample shown, 1 mg/mL PET was dispersed in an 18 mL reaction solution, resulting in 30.08% of PET successfully valorised to acetic acid upon irradiation. [M(CH_3COOH) = 60.05 g/mol; M($C_{10}H_8O_4$) = 192.17 g/mol]

$$OHCH_2CH_2OH \rightarrow CH_3COOH + H_2 \qquad (Eq.\ 5)$$

1. Amount of PET converted to acetic acid (in µmol) *(Eq. 6)*

$$\frac{concentration\ [mg/mL] * volume[mL]}{M(\mathrm{CH_3COOH})} = \mu mol\ PET\ converted$$

2. Conversion of µmol to mg *(Eq. 7)*

$$\frac{\mu mol\ PET\ consumed * M(\mathrm{C_{10}H_8O_4})}{1000} = mg\ PET$$

Table S10: Detected acetic acid concentration and amount of PET converted, shown in mg and %.

Sample	Concentration [mg/mL]	mg generated per 18mL reaction solution	µmol acetic acid = PET	PET consumption [mg]	PET consumption [%]
P25-Pt/70 °C	0.094	1.692	0.028	5.41	30.08

Supplementary note 7. PET conversion to gas phase products

The conversion efficiency of 18 mg of PET microplastic powder to the respective gaseous products (H_2, CH_4 and CO_2) was assessed by employing the equations described below (*Eq. 8* and *Eq. 9*). Hereby, the molecular formular of a single PET unit [$M(C_{10}H_8O_4)$ = 192,17 g/mol] was taken into account. The results are summarised in Table S11-S13.

1. Amount of PET converted to respective gaseous product (in µmol) *(Eq. 8)*

$$\frac{produced\ \mu mol * number\ of\ atoms\ of\ the\ generated\ molecule}{number\ of\ atoms\ per\ mol\ PET\ monomer} = \mu mol\ PET\ consumed$$

2. Conversion of µmol to mg *(Eq. 9)*

$$\frac{\mu mol\ PET\ consumed * M(C_{10}H_8O_4)}{1000} = mg\ PET$$

<u>Example: generated H_2 of P25-Pt/RT:</u>

(1) $\frac{14{,}325\ \mu mol\ H_2 * 2}{8\ \mu mol\ H/\ mol\ PET} = 3{,}58\ \mu mol\ PET\ converted\ to\ H_2$

(2) $\frac{3{,}58\ \mu mol\ PET\ consumed * 192{,}17\ g/mol}{1000} =$ 0,688 mg PET converted to H_2

Table S11: Calculated quantities of PET converted to H_2, presented in mg and %.

Hydrogen			
sample	H_2 [µmol]	PET consumption [mg]	PET consumption [%]
P25-Pt/RT	14,325	0,6882	3,823
P25/RT	0,006	0,0003	0,002
P25-Pt/70 °C	76,753	3,6874	20,486
P25/70 °C	0,357	0,0171	0,095

Table S12: Calculated quantities of PET converted to CH_4, presented in mg and %.

Methane			
sample	CH_4 [µmol]	PET consumption [mg]	PET consumption [%]
P25-Pt/RT	0,212	0,0041	0,023
P25/RT	0,055	0,0011	0,006
P25-Pt/70 °C	1,216	0,0234	0,130
P25/70 °C	0,174	0,0033	0,019

Table S13: Calculated quantities of PET converted to CO_2, presented in mg and %.

Carbon dioxide			
sample	CO_2 [µmol]	PET consumption [mg]	PET consumption [%]
P25-Pt/RT	0,068	0,0013	0,007
P25/RT	0,020	0,0004	0,002
P25-Pt/70 °C	0,009	0,0002	0,001
P25/70 °C	0,096	0,0018	0,010

Supplementary note 8. Solar-light-driven photoreforming

The feasibility in employing the solar spectrum was evaluated by irradiating two samples with a broad band Xe lamp (with IR filter). Hereby, a sample with P25-Pt and room temperature was set in contrast to neat P25 as photocatalyst and 70 °C. Both setups were irradiated for 24 hours. SEM images taken after the experiments are shown in Figure S8. Obtained yields of the gas phase products and calculated AQY values are summarised in Table S14 and S15. Moreover, the liquid phase of one of the samples was further measured via NMR spectroscopy (Figure S9).

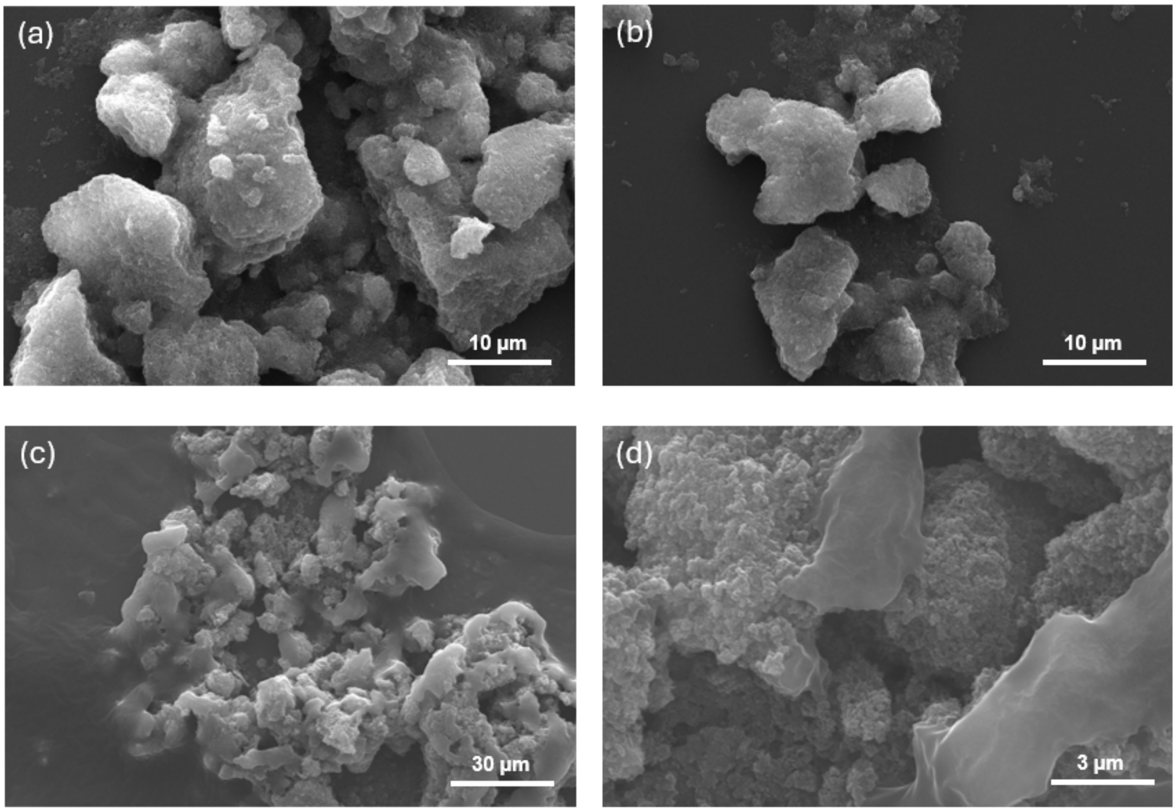

Figure S8: SEM images of the recovered powders: (a) and (b) PET after photoreforming with P25-Pt for 24 hours at RT and employing a broad band Xe lamp, (c) and (d) PET with P25 after irradiation for 24 hours at 70 °C and employing a broad band Xe lamp.

The sample irradiated at room temperature (Figure S8 a and b) shows adhesion of P25-Pt on the microplastic particles, similar to what is observed for upscaled sample under UV irradiation (Figure S12). However, the Xe light source appears to have resulted in less etching of the PET (i.e. reduced degradation). In contrast, the experiments carried out at 70 °C (Figures S8 c and d) illustrate some smooth surfaces in addition to the spherically shaped particles of the photocatalyst. Contrary to previous assumptions that elevated temperature aids the photoreforming process substantially, it seems that this parameter alone is not sufficient for the production of either gases when employing the solar spectrum, concluding in a mere 0.001 µmol/h of H_2. However, there appears to be more of a selectivity towards CH_4 generation, yielding twice the amount than H_2 (Table S14). Subsequent liquid phase analysis via NMR (Figure S9) and HPLC (Table S8) confirm the formation solely of the plastics' monomers - ethylene glycol and terephthalate. Only minor quantities of formic acid were attained (based on HPLC analysis) despite 16% of PET was successfully hydrolysed to ethylene glycol. In addition, NMR of this sample solely illustrates peaks corresponding to terephthalate and ethylene glycol in a ratio of around 1:1, equating to 0.159 mg/mL for the former (Figure S9). We conclude photoreforming was unsuccessful

under these conditions, presumably due to low conversion rates and insufficient intensity of incoming light. This observation is again further validated by the SEM images, displaying less degradation and more decomposition of PET in its monomers, as indicated by the described pale and smoother surfaces.

Table S14: Detected quantities of H_2 and CH_4 during experiments conducted with a broad brand Xe lamp to simulate the solar spectrum.

Hour	H_2 [µmol/h]	CH_4 [µmol/h]
P25-Pt/RT/Xe lamp	0.209	0.009
P25/70 °C/Xe lamp	0.001	0.002

Table S15: Calculated AQY values in % derived from the H_2 values summarised in Table S14.

sample	AQY [%]
P25-Pt/RT/Xe lamp	0.84
P25/70 °C/Xe lamp	0.00373

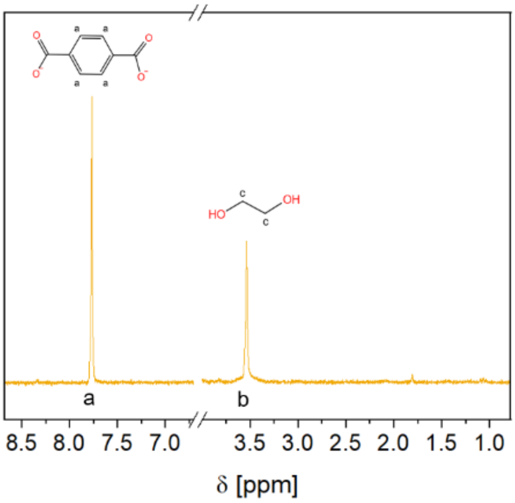

Figure S9: ^{1}H NMR spectrum of P25 at 70 °C (inert, under Xe lamp), measured in D_2O and 1M NaOH (1:10), with water suppression.

Supplementary note 9. Window sill experiments

To investigate the feasibility of the process under ambient and natural conditions, four samples (see Table S16 and Figure S10) were prepared, containing microplastic powder (1 mg/mL) (*Sample 1* and *Blank 1*) or a piece of commercial PET bottle (1 cm x 1 cm) (*Sample 2* and *Blank 2*). The substrates were dispersed in 1M NaOH, with or without P25-Pt photocatalyst (0.5 mg/mL). Each sample was purged with He for 10 minutes, then sealed with parafilm and nail varnish before placing them on a balcony for around 20 months, investigating the impact of natural sun light in the course of all four seasons. Subsequently the headspace was measured via GC. Results indicate the generation of 0.165 µmol H_2 for *Sample 1*. Conversely, no H_2 was measured for *Sample 2*. While we cannot ensure that the absence of H_2 produced from a piece of commercial PET is not related to the poorly sealed vial, it is important to note successful production of H_2 in the microplastic-containing vial. Both prepared blanks did not result in H_2, emphasizing the necessity of a photocatalyst for the photocatalytic reaction to proceed.

Table S16: Overview of window sill experiments conducted over the course of 20 months.

Sample	substrate	photocatalyst	H_2 [µmol]
Sample 1	Microplastic powder	P25-Pt	0.165
Blank 1	Microplastic powder	X	0
Sample 2	Plastic bottle	P25-Pt	0
Blank 2	Plastic bottle	X	0

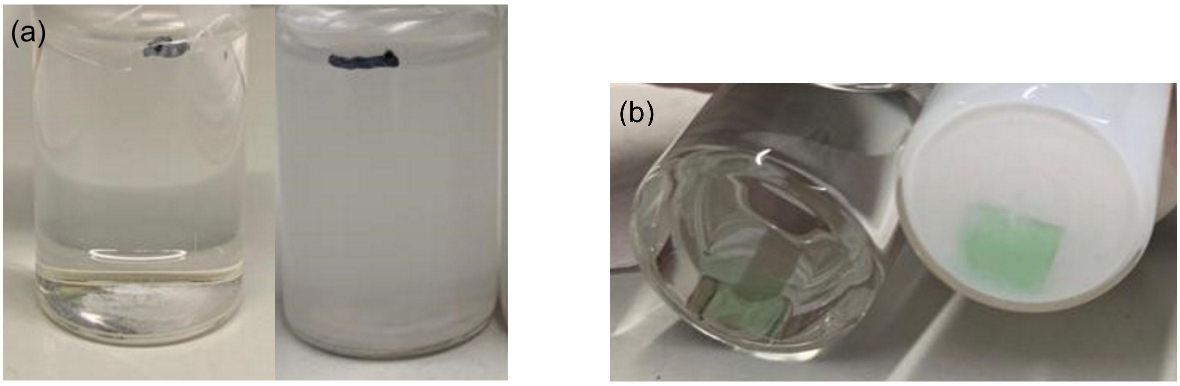

Figure S10: Prepared samples for the window sill experiments. (a) Blank 1 (left) and Sample 1 (right) (b) Blank 2 (left) and Sample 2 (right).

Supplementary note 10. PET degradation mechanism

Zeta potential measurements were performed to elucidate on the surface charge values of the PET and catalyst suspensions to shed light on the possible interactions between the solids in the reaction medium. Results are illustrated in Figure S11 and Table S17.

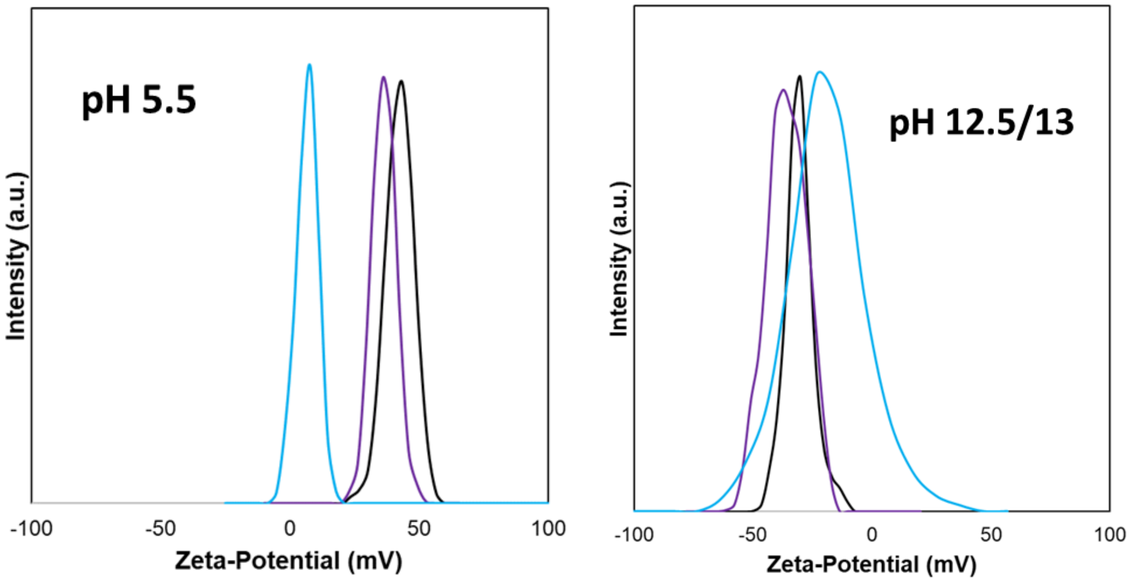

Figure S11: Zeta potential measurement of PET (blue), P25 (black) and P25-Pt (purple) at pH 5.5 (left) and pH 12-13 (right).

Table S17: Summary of zeta potential measurement results at given pH.

Sample	Average Zeta-Potential	Standard Deviation	Measured pH
1 mg mL^{-1} PET in nano-pure water	5.8 mV	0.76	5.86
0.5 mg mL^{-1} P25 in nano-pure water	32.1 mV	0.55	5.68
0.5 mg mL^{-1} P25-Pt in nano-pure water	26.4 mV	0.53	5.29
1 mg mL^{-1} PET in 0.2 M NaOH	-23.7 mV	2.83	13.32
0.5 mg mL^{-1} P25 in 0.01 M NaOH	-33.1 mV	1.78	12.48
0.5 mg mL^{-1} P25-Pt in 0.01 M NaOH	-37.9 mV	3.53	12.45

Photoluminescence spectroscopy (PL) was carried out to elucidate the photocatalytic mechanism of the prepared P25-Pt photocatalyst during the photoreforming of PET. Generally, photocatalytic oxidation can occur through either generated holes upon excitation (direct pathway) or via hydroxyl radicals ($^{\cdot}OH$) and other reactive oxygen species (indirect pathway). To elaborate on the underlying mechanism of the investigated system, the addition of the PET to the reaction mixture was compared to a blank experiment and the effect of air in contrast to inert conditions (He) of the reaction atmosphere was additionally investigated. Terephthalic acid (0.003 M) was added as a radical trapping agent to the 1M NaOH reaction medium and irradiation took place for 5 hours. The emission spectra obtained in deaerated solutions depict the characteristic emission peak of 2-hydroxyterepthalic acid centred at 430 nm,

resulting from the presence of ·OH and their reaction with terephthalic acid. In both cases, i.e. under anaerobic (Figure 4c) and oxygen-rich conditions (Figure S12), the presence of PET in the reaction mixture decreases peak intensity at 430 nm, suggesting that PET either consumes ·OH directly from the solution, or it can be oxidized directly via holes generated by TiO_2. The addition of oxygen to the reaction medium leads to a reduced disparity between the PET-containing sample and blank (Figure S12 vs Figure 4c). Since O_2 can generate superoxide radicals ($O_2^{\cdot-}$) by reduction via electrons photoexcited in TiO_2, they are likely to compete with ·OH for PET oxidation and dominate it based on this result.

Additionally, a screening of different quenchers was carried out to further investigated present radical species. Herein, 1mM of isopropanol (IPA), triethanolamine (TEOA), 1,4-benzoquinone (BQ), Na_2EDTA, and $CHCl_3$ were added to the reaction solution. These compounds have been reported to act as selective quenchers/traps for a specific set of radical species – IPA as ·OH trap. TEOA and EDTA as h^+ scavengers and BQ and $CHCl_3$ as $O_2^{\cdot-}$ quenchers.[5] In a single experiment, a given quencher (1 mM) was added to the 1M NaOH solution of P25-Pt prior to illumination. The quenching behaviour was evaluated by comparing the resulting H_2 and CH_4 production activities with and without the presence of quenchers. Figure S13 and Table S18 summarise quenching behaviours observed with different quenchers for both H_2 and CH_4 production with respect to the quencher-free-activity. Interestingly, only BQ resulted in a strong activity drop (99.7% less H_2 and 94.3% less CH_4 was produced in its presence), while $CHCl_3$ showed some selective quenching, diminishing H_2 yield by around 30%. Interestingly, the other employed quenchers only appeared to aid solar fuel generation (resulting in 2-7 times higher activity), which we assign to them additionally acting as sacrificial electron donors. Given the prime role of BQ and $CHCl_3$ in trapping $O_2^{\cdot-}$ [6,7], and in light of the PL studies (see Figure 4c and S12) that confirmed direct hole transfer mechanism (also collaborated by SEM images of damaged PET particles, see Figure 4d - f), we can conclude that $O_2^{\cdot-}$ and direct oxidation via photoexcited h^+ are the main contributors to the degradation mechanism.

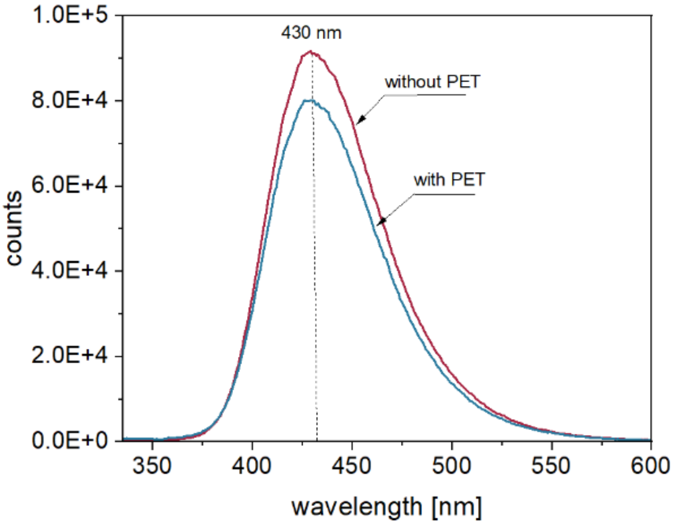

Figure S12: Emission spectrum of PET photoreforming with P25-Pt (1 wt%) under aerobic conditions. The addition of terephthalic acid leads to the formation of 2-hydroxyterepthalic acid when ·OH are present. (λ_{exc} = 315 nm. λ_{em} = 430 nm).

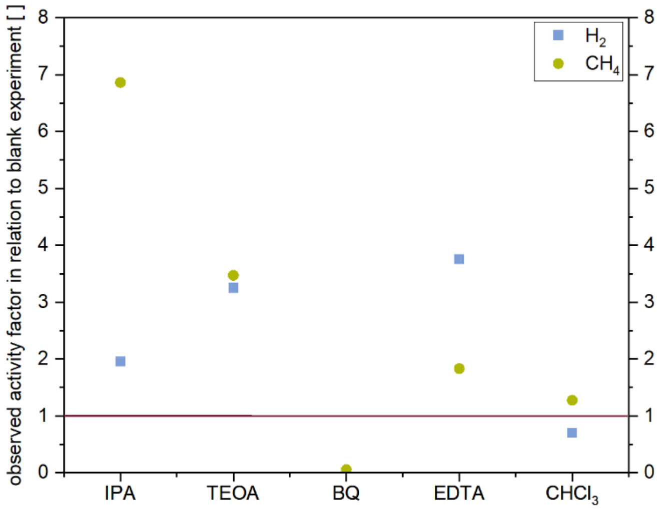

Figure S13: Quenching of H_2 and CH_4 generation resulting from IPA, TEOA, BQ, EDTA and $CHCl_3$ addition in contrast to standard experiments (y-axis = 1). The tests were performed at 1 h irradiation with 365 nm using P25-Pt at RT.

Table S18: Measured H_2 and CH_4 activities from the blank (1M NaOH) in comparison to the respective quencher, shown in µmol/h.

sample	H_2 [µmol/h]	CH_4 [µmol/h]
Blank	2,515	0,04643
IPA	4,925	0,31855
TEOA	8,181	0,16126
BQ	0,008	0,00264
EDTA	9,447	0,0851
$CHCl_3$	1,776	0,05927

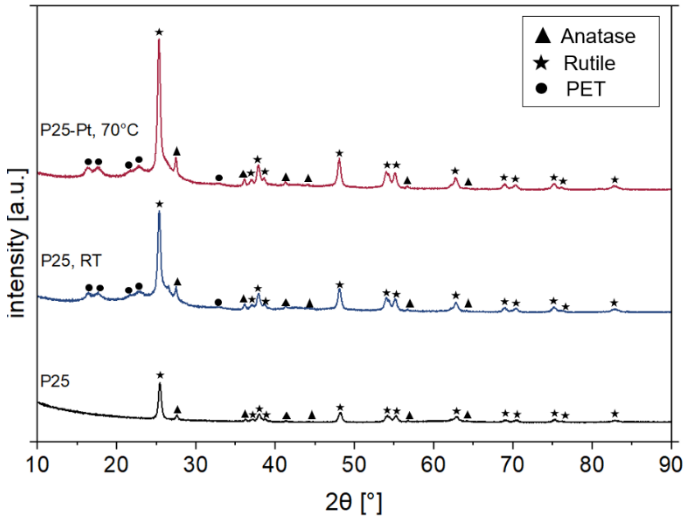

Figure S14: XRD pattern of solids recovered after 5h photoreforming of PET and neat P25 as reference.

XRD pattern of the recovered solid (Figure S14) displays the characteristic peaks of rutile and anatase phases of P25 (suggesting the catalyst's stability), along with additional peaks corresponding to unprocessed PET. No unexpected phase change has been observed, in line with stable catalytic performance.

Supplementary note 11. Upscaled experiment

To elaborate on commercial feasibility, a set of experiments was carried out with 100 mL 1M NaOH in a round bottom flask, 1 mg/mL PET and 0.5 mg/mL P25-Pt, irradiated with a 365 nm light source at room temperature and for 32 hours. SEM images were taken after illumination (Figure S15). In the course of this upscaled experiment, the gaseous phase was measured every hour via GC within the first 5 hours and every two hours from hour 24 to 32. The quantities of generated solar fuels are summarised in Table S19.

Table S19: Detected quantities of H_2 and CH_4 during the upscaled experiment using P25-Pt (at RT).

Sampling	H_2 [µmol/h]	CH_4 [µmol/h]
1	19.57	0.008
2	6.04	0.194
3	13.98	0.148
4	7.77	0.243
5	14.76	0.142
Production rate Day 1	10.35	0.197
24	10.44	0.175
26	8.03	0.161
30	13.68	0.237
32	8.48	0.199
Production rate Day 2	8.35	0.160

For the upscaled experiment comprised of P25-Pt and irradiated at RT (Figure S15a and b), the PET fragments are covered in spherically shaped photocatalyst particles. However, it is clear that UV irradiation is more successful in terms of degradation of the microplastic as more of an etching is visible (comparing to Figure S8a and b above). It has previously been established that the energy provided by UV photons is adequate for the cleavage of C-C and C-H bonds, thus further facilitating the oxidation of the substrate.[8]

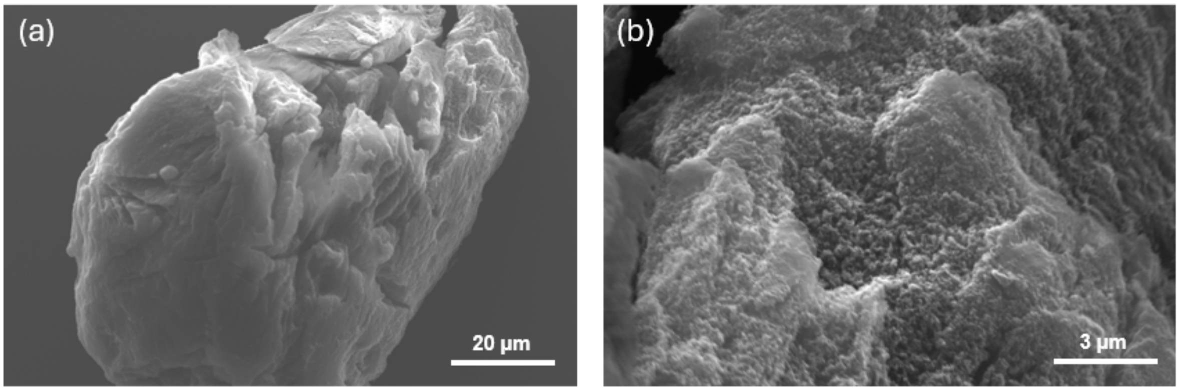

Figure S15: (a) and (b) SEM images of the PET particles after irradiation for 32 hours in an upscaled manner with P25-Pt and at RT.

Supplementary note 12. Images of the reaction solutions

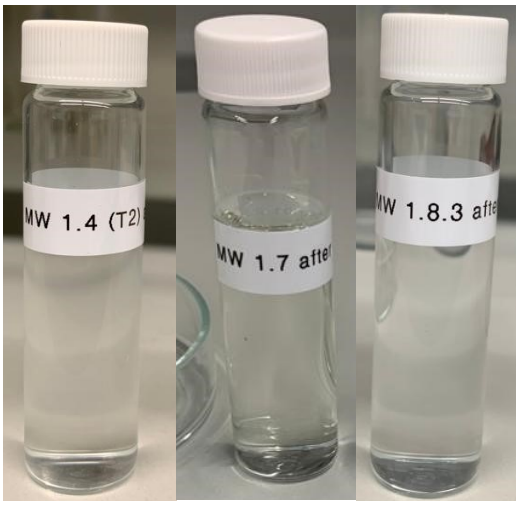

Figure S16: Filtered solutions of P25/RT (left), P25-Pt/70 °C (middle) and P25/70 °C (right). In comparison to the other two samples, P25-Pt/70 °C demonstrates a slightly pale yellow in colour.

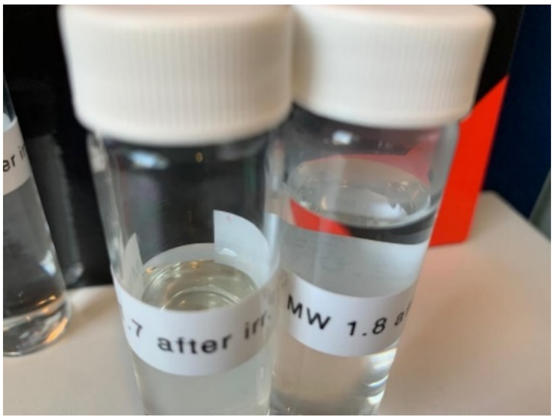

Figure S17: P25-Pt/70 °C (left) resulted in a pale yellow solution after 5h irradiation, whilst P25/70 °C (right) remained transparent, as most of the other samples.

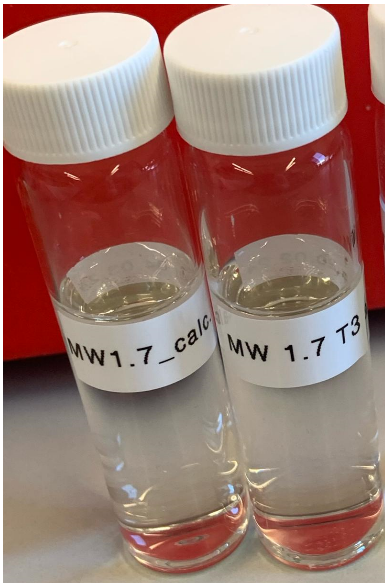

Figure S18: This image illustrates no visible impact of TiO_2 calcination on solution colour, depicting P25-Pt/70 °C/calcined (left) and P25-Pt/70 °C (right).

References

1 S. Batool, J. S. Schubert, P. Ayala, H. Saito, M. J. Sampaio, cd S. Eliana Da Silva, cd G. Cí audia Silva, cd L. Joaquim Faria, cd Dominik Eder and A. Cherevan, *Sustainable Energy Fuels*, 2024, **8**, 1225.

2 M. Qureshi and K. Takanabe, DOI:10.1021/acs.chemmater.6b02907.

3 L. Wang and J. Yu, *Interface Science and Technology*, 2023, **35**, 1–52.

4 K. S. P. Karunadasa and C. H. Manoratne, *J Solid State Chem*, 2022, **314**, 123377.

5 S. N. Myakala, M. Ladisich, P. Ayala, H. Rabl, S. Batool, M. S. Elsaesser, A. Cherevan and D. Eder, *J. Mater. Chem. A*, 2024, **12**, 207890.

6 F. Puga, J. A. Navío and M. C. Hidalgo, *Appl Catal A Gen*, DOI:10.1016/j.apcata.2024.119879.

7 M. Zhu, J. Lu, Y. Hu, Y. Liu, S. Hu and C. Zhu, *Environmental Science and Pollution Research*, 2020, 31289–31299.

8 F. Zhang, Y. Zhao, D. Wang, M. Yan, J. Zhang, P. Zhang, T. Ding, L. Chen and C. Chen, *J Clean Prod*, DOI:10.1016/J.JCLEPRO.2020.124523.
